# Benzbromarone, Quercetin, and Folic Acid Inhibit Amylin Aggregation

**DOI:** 10.3390/ijms17060964

**Published:** 2016-06-18

**Authors:** Laura C. López, Olga Varea, Susanna Navarro, José A. Carrodeguas, Natalia Sanchez de Groot, Salvador Ventura, Javier Sancho

**Affiliations:** 1 Departamento de Bioquímica y Biología Molecular y Celular, Facultad de Ciencias, Universidad de Zaragoza, Pedro Cerbuna 12, 50009 Zaragoza, Spain; lclopez@unizar.es (L.C.L.); ovarea@bifi.es (O.V.); carrode@unizar.es (J.A.C.); 2 Biocomputation and Complex Systems Physics Institute (BIFI), Joint Unit BIFI-IQFR (CSIC), Mariano Esquillor s/n, Edificio I + D, 50018 Zaragoza, Spain; 3 Institut de Biotecnologia i Biomedicina and Departament de Bioquímica i Biologia Molecular, Universitat Autònoma de Barcelona, 08193 Bellaterra, Spain; Susanna.Navarro.Cantero@uab.cat (S.N.); nsdgroot@gmail.com (N.S.d.G.); 4 Aragon Health Research Institute (IIS Aragón), Universidad de Zaragoza, 50009 Zaragoza, Spain

**Keywords:** quercetin, benzbromarone, folic acid, amylin, amyloid, aggregation, type II diabetes

## Abstract

Human Amylin, or islet amyloid polypeptide (hIAPP), is a small hormone secreted by pancreatic β-cells that forms aggregates under insulin deficiency metabolic conditions, and it constitutes a pathological hallmark of type II diabetes mellitus. In type II diabetes patients, amylin is abnormally increased, self-assembled into amyloid aggregates, and ultimately contributes to the apoptotic death of β-cells by mechanisms that are not completely understood. We have screened a library of approved drugs in order to identify inhibitors of amylin aggregation that could be used as tools to investigate the role of amylin aggregation in type II diabetes or as therapeutics in order to reduce β-cell damage. Interestingly, three of the compounds analyzed—benzbromarone, quercetin, and folic acid—are able to slow down amylin fiber formation according to Thioflavin T binding, turbidimetry, and Transmission Electron Microscopy assays. In addition to the *in vitro* assays, we have tested the effect of these compounds in an amyloid toxicity cell culture model and we have found that one of them, quercetin, has the ability to partly protect cultured pancreatic insulinoma cells from the cytotoxic effect of amylin. Our data suggests that quercetin can contribute to reduce oxidative damage in pancreatic insulinoma β cells by modulating the aggregation propensity of amylin.

## 1. Introduction

Type II diabetes is a metabolic disorder of increasing prevalence (10% in USA and 5% worldwide) [1,2]. It is characterized by resistance to and/or low levels of insulin production linked to the damage of pancreatic β cells [3,4]. This damage has been associated with the presence of amyloid aggregates that become more abundant as the disease progresses [5,6]. The major component of β cells amyloid deposits is a 37 amino acid peptide, termed human islet amyloid polypeptide (hIAPP) or amylin, a hormone secreted along with insulin in the pancreas [7]. Amylin contributes to the regulation of blood glucose levels by controlling the rate at which glucose enters the blood stream. Amyloid fibers of IAPP have been described in humans, other primates, and cats [8]. Rodent IAPP, only differing from hIAPP in six residues, does not form amyloids [9]. It is believed that hIAPP amyloidogenicity may be related to a protein segment comprising amino acids 20–39 [10].

Proteins can form different types of insoluble aggregates, either amorphous or fibrillar. Amylin forms stacked cross-β-sheet fibers [11,12,13,14]. Several studies have pointed out that in amyloid diseases the main cytotoxic effect is associated with the small early forming oligomers of misfolded proteins rather than with the larger mature amyloid fibers [15,16,17,18,19,20]. Thus, inhibiting the formation of cytotoxic oligomers early in the aggregation reaction might constitute a promising strategy to minimize cellular damage in type II diabetes and in other amyloidogenic diseases [21], such as Alzheimer’s or Parkinson’s disease, where aggregates of the amyloid β peptide, Tau, or α-synuclein seem to play major roles [22,23]. Indeed, the inhibition of amyloid β peptide aggregation and Tau protein by small chemical compounds has already reached clinical trials [24,25,26,27].

We have previously shown the feasibility of identifying bioactive compounds by screening large libraries of chemicals [28,29,30,31,32]. Indeed, this is a very effective approach to rapidly find leads for further improvement. Here we have searched for inhibitors of amylin aggregation using an amylin fragment comprising amino acids 8–37, which includes all known amylin amyloid determinants [33] and is known to induce cytotoxicity in pancreatic β-cells [34]. We have found three well-known compounds—folic acid, quercetin, and benzbromarone—that inhibit amylin aggregation *in vitro* and we have tested their effects on amylin treated pancreatic rat insulinoma β-cells.

## 2. Results and Discussion

### 2.1. Identification of Inhibitor Candidates

**Figure 1:**
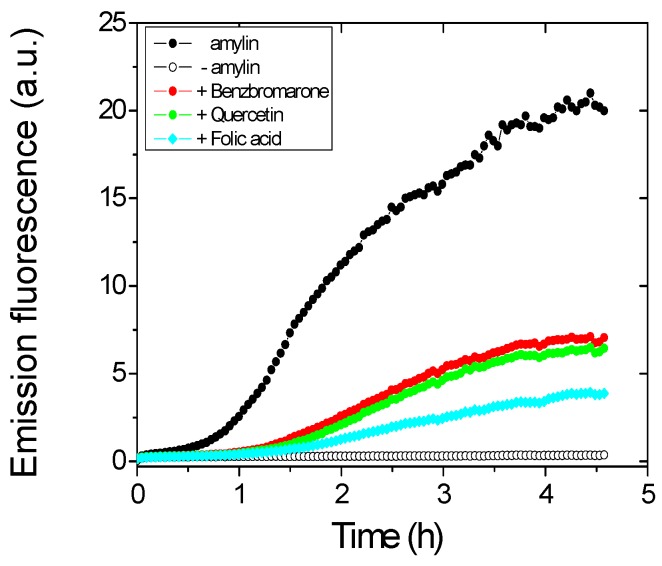
Inhibition of amylin (8–37) aggregation by benzbromarone, quercetin, and folic acid, followed by an increase in Thioflavin T (ThT) emission fluorescence at 482 nm. Black dots: positive aggregation control, only amylin; white dots: negative aggregation control (no amylin); Red, green, and cyan dots: aggregation of amylin in presence of benzbromarone, quercetin, and folic acid, respectively.

To identify inhibitors of amylin (8–37) aggregation, a high-throughput (HTS) assay based on the increase in fluorescence associated to Thioflavin T (ThT) binding to amyloid fibrils was used [35]. Typical of an amyloidogenic peptide, Amylin (8–37) aggregation kinetics in the presence of ThT follows a sigmoidal curve that reflects a nucleation-dependent growth mechanism, showing an initial nucleation step followed by an elongation phase ending in a stationary phase (Figure 1). The Prestwick library of 1220 compounds was first screened for inhibitors of aggregation including mixtures of five different chemicals in each well. Then, compounds present in positive wells where the aggregation of amylin (8–37) was inhibited were subsequently evaluated individually using otherwise identical assay conditions. In this way, apparent inhibitory compounds were identified. Some of them turned out to be true inhibitors and some were false positives that could be discarded with further analysis (see below). Figure 1 shows the effects on the aggregation kinetics of the three chemical compounds that were finally selected among the inhibitory candidates initially identified using the ThT assay. When any of those compounds were present in the solution the increase in ThT fluorescence observed was lower than that corresponding to a solution of peptide and ThT in the absence of the compound. Detecting active compounds using fluorescence-based assays is prone to produce false positives, especially among compounds absorbing the light emitted by the fluorescence probe used, because such compounds lower the emission intensity even if they do not inhibit aggregation. In order to distinguish between true inhibitors and false positives, turbidity and transmission electron microscopy (TEM) analyses were performed.

### 2.2. Identification of True Inhibitors by Turbidity and Transmission Electron Microscopy (TEM) Analyses

**Figure 2:**
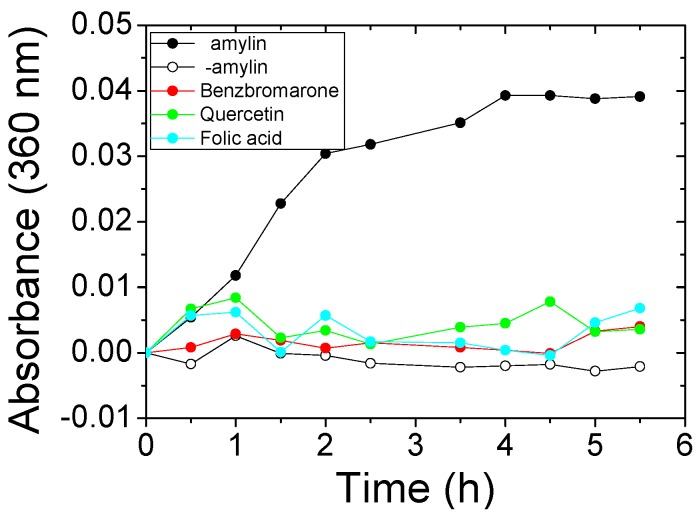
Kinetic aggregation assays of amylin (8–37) in the presence of benzbromarone, quercetin, and folic acid, followed by solution turbidity. Turbidity was measured as absorbance at 360 nm. Black dots: positive aggregation control (only amylin); white dots: negative aggregation control (no amylin); Red, green, and cyan dots: aggregation of amylin with the addition of benzbromarone, quercetin, and folic acid, respectively.

**Figure 3:**
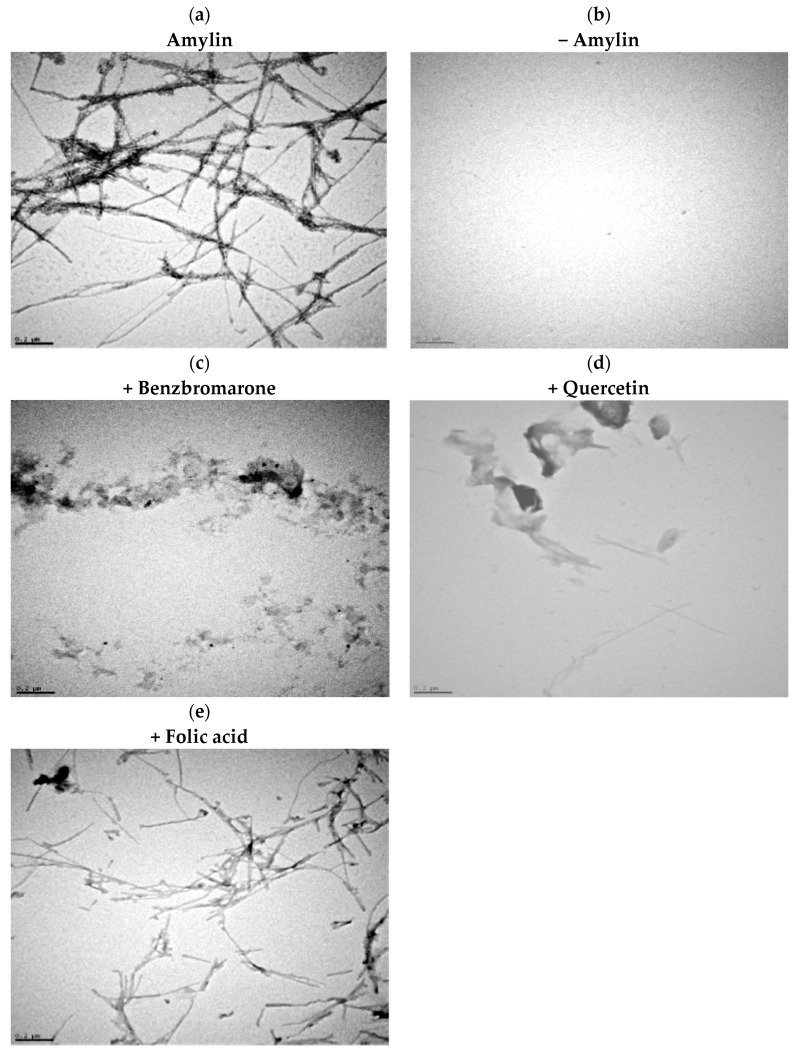
Amylin aggregation inhibition visualized using transmission electron microscopy (TEM). Amylin (8–37) at 50 µM was incubated in the presence or absence of the compounds (at 100 µM), fibers were negatively stained and imaged using TEM. Positive control (amylin alone): panel (**a**); negative control (no amylin): panel (**b**); amylin in presence of benzbromarone, quercetin, and folic acid: panels (**c**, **d**, or **e**), respectively. The length of the scale bars is 0.2 µm. Compounds strongly inhibit amylin amyloid fibril formation.

A simple turbidimetric method was used to find out which of the compounds initially identified in the ThT fluorescence assay were true aggregation inhibitors and which ones were false positives. Aggregation of amylin (8–37) gives rise to high molecular weight aggregates that scatter light and increase the turbidity of the solution, which can be detected by measuring the increase in absorbance at 360 nm. For this assay, amylin (8–37) was incubated in the presence of each candidate compound for 5.5 h, and the absorbance at 360 nm was measured every 30 min. The kinetics observed for the aggregation of amylin controls in the absence of an inhibitor are compared to those observed when true inhibitors were present (Figure 2). Three compounds completely avoided the building of turbidity in the amylin solution and were considered to be aggregation inhibitors. This was confirmed using TEM by studying the influence of the inhibitors in the formation of amylin fibers. Amylin self-assembles into typical long, unbranched fibrils as well as fibril bundles and twisted ribbons with various lengths (up to 2 mm) (Figure 3a). Two of the inhibitors significantly lowered the amount of fibers formed (Figure 3c,d). In the presence of compound 3 fibers are still detectable, but they differ in morphology from those formed in its absence, indicating that the compound also impacts the fibrillation process (Figure 3e).

The influence of Congo red and methylene blue in amylin fibril formation was also tested, as they have been described to protect against amylin cytotoxicity. When those compounds were first shown not to impair amylin aggregation [36,37], it was hypothesized that either their binding to the fibrils blocked their toxic effect or that the compounds inhibited the accumulation of toxic oligomeric species by promoting fibrillation. We have incubated amylin in the presence of Congo red or methylene blue in the same conditions used for our three inhibitory compounds and observed that they reduce amylin solution light scattering (Figure S1a) and, in agreement with previously described observations [36,37], well-structured amylin fibrils are clearly observed in the presence of either molecule (Figure S1b).

### 2.3. Chemical Nature and Cellular Toxicity of the Three Inhibitors

The chemical structures of the three inhibitors are shown in Table 1. They are heterocyclic compounds exhibiting no obvious chemical relationship: benzbromarone (molecular mass (MM) = 424.1 and logP = 6.0), quercetin dihydrate (MM = 338.27 and logP = 1.5), and folic acid (MM = 441.4 and logP = −0.99). Folic acid contains one asymmetric center while benzbromarone and quercetin are not chiral.

The toxicity of the three inhibitors at different concentrations ranging from 10 nM to 200 µM was tested on HeLa cells using the Cell Proliferation Kit II (Roche, Basel, Switzerland), which detects cell dehydrogenase activity, by transformation of XTT (a tetrazolium salt) into a soluble colored formazan that can be quantified by absorbance. This absorbance is proportional to the number of viable cells. Toxicity curves (Figure S2) were fitted to a dose response equation to calculate half inhibitory concentrations (IC_50_). The most toxic inhibitor was benzbromarone, (IC_50_ = 43 ± 7 µM), then quercetin (110 ± 33 µM), although both of them were toxic only at relatively high concentrations. The toxicity of folic acid was very low (>200 µM) and could not be determined since it was above the tested range. These results showed that benzbromarone, quercetin, and folic acid display no significant toxicity towards HeLa cells at the medium-low concentration range tested. We then decided to keep a concentration of 10 µM for the three compounds during the following cytotoxicity protection experiments on cultured cells.

### 2.4. Partial Inhibitory Effect of Quercetin on the Cytotoxicity Induced by Amylin (8–37) in Rat Insulinoma Pancreatic (RIN-m5F) Cells

Rat insulinoma pancreatic cells (RIN-m5F) were used to evaluate whether any of the aggregation inhibitors could protect them against the cytotoxic effect exerted by amylin. The dyes Congo red and methylene blue, which have been previously described to protect against amylin cytotoxicity [36,37], were also tested for comparison. RIN-m5F cells were first treated for 48 h with inhibitors (see Materials and Methods) and cell viability was assessed by the XTT assay. Cell viability variation due to the compounds themselves was discarded since at the concentration used, none of the three newly discovered inhibitors, nor Congo red, reduced cell viability relative to the control. However, methylene blue did exert toxicity and it was not further tested.

**Figure 4:**
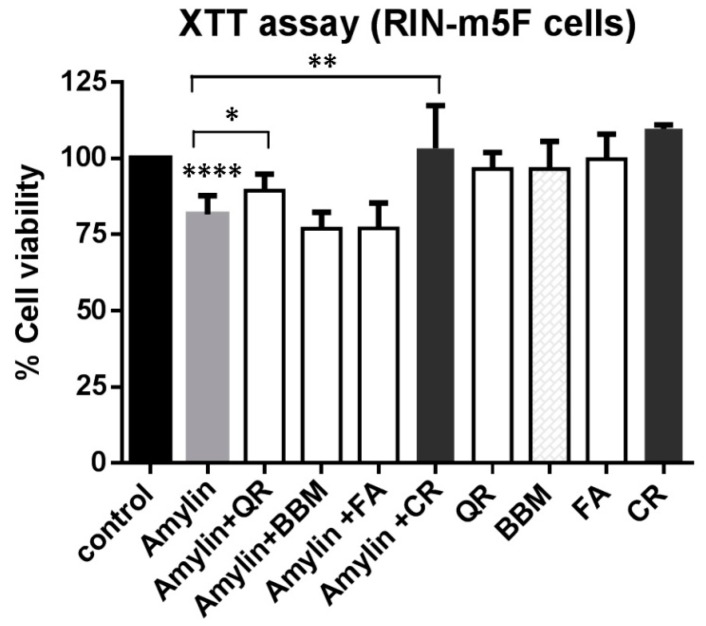
Quercetin reduction of amylin (8–37) cytotoxicity in pancreatic rat insulinoma Rat insulinoma pancreatic (RIN-m5F) β-cells. Cells were treated for 48 h with amylin (75 µM) in the presence or absence of benzbromarone (BBM, 10 µM), quercetin (QR, 10 µM), folic acid (FA, 10 µM), or Congo red (CR, 100 µM), and cell viability was measured using a 2,3-Bis(2-methoxy-4-nitro-5-sulfophenyl)-2H-tetrazolium-5-carboxanilide: XTT assay. The results obtained were normalized to control cells (dimethyl sulfoxide: DMSO treated) and expressed as mean values ± SD (*n* = 7 independent experiments). No statistical differences were obtained between the conditions where only the compounds were added and the control. Statistical analysis was carried out using an unpaired *t* test. * *p* < 0.05; ** *p* < 0.01; **** *p* < 0.0001.

In our assay, cells treated with 75 µM human amylin (8–37) for 48 h showed (Figure 4) an 18% reduction in cell viability, compared to control cells (*p* = 0.0001). Neither benzbromarone nor folic acid could protect the cells from the cytotoxic effect exerted by amylin. However, quercetin increased the viability of amylin-challenged RIN-m5F cells from 81% to 89% (*p* = 0.028). While the statistical significance of this effect is clear, its magnitude is small. Accordingly, evaluation of the dose response quercetin protective effect will require the development of a more sensitive assay. On the other hand, and as expected from existing data, Congo red fully recovered the viability of amylin challenged RIN-m5F cells (*p* = 0.0076).

### 2.5. Some Previous Evidences Relating Benzbromarone, Quercetin or Folic Acid with Type II Diabetes

The three inhibitory compounds found—benzbromarone, quercetin, and folic acid—have been widely studied in pharmacological or clinical contexts. The observation that they inhibit amylin aggregation *in vitro* has prompted us to examine whether they have been previously reported to exert a beneficial influence in type II diabetes. Benzbromarone is a uricosuric agent used to treat gout [38]. In a non-exhaustive literature search we have only found circumstantial evidence relating benzbromarone with glucose blood levels [39]. On the other hand, folic acid (vitamin B_9_) is an important biological cofactor necessary for DNA synthesis. Folic acid has been proposed to be beneficial in type II diabetes due to its roles in accelerating the hydrolysis of fats, in lowering homocysteine levels [40], or in improving the endothelial function [41]. Our results suggest a possible additional role of folic acid, namely in reducing amylin aggregation, which might be worth testing. Quercetin is a flavonoid found in many fruits, vegetables, and red wine. Interestingly, there is a wealth of reports indicating that quercetin exhibits numerous beneficial effects in diabetes [42,43]. One such effect appears to be that of regenerating pancreatic islets and preserving the integrity of pancreatic β cells [44,45,46,47], and one mechanism invoked to explain this effect is that of reducing oxidative stress. Quercetin has also been described to contribute to the control of blood glucose levels *in vivo* in animals with streptozotocin-induced diabetes [44,45,48,49] and to improve hyperglycemia, dyslipidemia, and antioxidant status in an animal model of type II diabetes mellitus: C57BL/KsJ-*db*/*db* mice [50]. Clinical trials to test effects of quercetin on blood sugar and blood vessel function in type II diabetes have been conducted [51]. In a recent study, morin, a flavonoid closely related to quercetin, has been described to inhibit the aggregation of amylin and even to disaggregate its fibers [52], while quercetin has been reported not to inhibit amylin aggregation. Our results, however, clearly indicate that quercetin inhibits amylin aggregation (Figure 1, Figure 2 and Figure 3) and partly protects RIN-m5F cells from the damage exerted by extracellularly added amylin. The different experimental conditions employed in both studies might well account for this discrepancy; indeed, in the same study myricetin, a flavonoid closely related to quercetin, was found to be inactive against amylin aggregation, whereas another study has described its ability to interfere with amylin amyloid formation and cytoxicity [53]. In addition, quercetin has been shown to inhibit the aggregation of a number of unrelated amyloidogenic proteins linked to human disease, including the amyloid β peptide [54], lysozyme [55], insulin [56], and α-synuclein [57,58]. In the light of this reported generic anti-amyloidogenic activity and the data in the present study, we suggest that a more detailed examination of a potential beneficial effect of quercetin in type II diabetes by reducing amylin aggregation *in vivo* and its concomitant oxidative damage in β cells may be timely.

## 3. Materials and Methods

### 3.1. Materials

Human amylin (8–37) with a purity greater than 98% (as indicated by HPLC analysis) was acquired from the American Peptide Company, Inc. (Sunnyvale, CA, USA). A 1 mg/mL peptide stock solution was prepared by dissolving lyophilized peptide in 1,1,1,3,3,3-hexafluoro-2-propanol. The solution was aliquoted and lyophilized. When required, aliquots were dissolved in 10 mM sodium acetate (pH 5.5) to a final concentration of 500 µM, and centrifuged at 14,000 rpm in a microfuge for 30 min at 4 °C just before use. A library of 1120 approved drugs with purities greater than 90% was purchased from Prestwick Chemical (Strasbourg, France). Thioflavin T (ThT) was purchased from the American Peptide Company, Inc.

### 3.2. High Throughput Screening

ThT fluorescence increases upon binding to amyloid fibers [35]. A 650 µM ThT stock was prepared in Phosphate-buffered saline: PBS buffer. A ThT binding-based initial screening of the commercially available Prestwick library was performed using 96-well plates. Each well contained, in a total volume of 200 µL, 10 mM sodium acetate (pH 5.5), 50 µM amylin (8–37), 6.5 µM ThT and five different compounds (100 µM each). ThT emission fluorescence was continuously recorded with a FluoDia T 70 fluorimeter (PTI, Edison New Jersey, USA) using excitation and emission wavelengths of 450 and 500 nm, respectively. Amylin aggregation kinetics were followed at 37 °C for 4.5 h, with measurements every 2.5 min and 3-s agitation before each measurement.

### 3.3. Turbidity Test

Turbidity tests were used to identify false positives of the high throughput screening [31]. For this purpose, 50 µM amylin (8–37) was mixed in 10 mM sodium acetate (pH 5.5) with 100 µM compound in a 1-cm quartz cuvette. Aggregation kinetics were monitored in a spectrophotometer recording absorbance at 360 nm, every 30 min, for 5.5 h.

### 3.4. Transmission Electron Microscopy

Inhibitory compounds that passed the turbidity assay were further tested by transmission electron microscopy (TEM). The size and morphology of the amyloid fibrils formed by amylin (8–37) in the presence or absence of the different compounds were evaluated using a Hitachi H-7000-75 kV microscope (Hitachi, Tokyo, Japan). For this we used a fresh mixture of 50 µM Amylin (8–37) and 100 µM compound in 10 mM sodium acetate buffer (pH 5.5) with 2.5% DMSO. The samples were incubated at 37 °C for 24 h before a 5 µL drop of sample was placed on Formvar carbon support film on a copper grid. The samples were stained with 2% uranyl acetate for 1 min. Excess stain was removed and the samples were allowed to dry at room temperature.

### 3.5. Cytotoxicity Assays of Aggregation Inhibitors in HeLa Cells

The cytotoxicity of the three inhibitory compounds found by ThT screening and confirmed by turbidimetry and by TEM analysis was evaluated using HeLa cells growing in a RPMI-1640 medium with phenol red supplemented with 100 U/mL penicillin, 100 µg/mL streptomycin sulfate, and 10% fetal calf serum (all reagents from Invitrogen, Waltham, MA USA) at 37 °C in a 5% CO_2_ atmosphere. Cells were cultured in 25 cm^2^ polystyrene tissue culture flasks and sub-cultured every three days. To evaluate the toxicity of the compounds, 100-µL aliquots of culture medium containing 3 × 10^4^ cells were added to each well in the 96-well plates. After incubation for 24 h in the absence or presence of compounds at different concentrations (between 10 nM and 200 µM), cell viability was assessed using XTT: 2,3-Bis(2-methoxy-4-nitro-5-sulfophenyl)-5-[(phenyl-amino) carbonyl]-2h-tetrazolium hydroxide following the manufacturer’s instructions (Cell Proliferation Kit II, Roche). The culture medium was replaced with 100 µL of RPMI-1640 without phenol red plus 50 µL of XTT reagent. The plates were incubated at 37 °C for 4 h and the optical density at 450 nm was quantified using a spectrophotometric plate reader with the reference filter set to 620 nm. The values obtained for controls corresponding to samples without compounds were considered to represent 100% viability. Half inhibitory concentrations (IC_50_) were calculated for each of the three compounds evaluated by adjusting the viability obtained for the different concentrations of each compound, using a dose-response function implemented in Origin Pro ^®^ 8 (Northampton, MA, USA).

### 3.6. Cytotoxicity Assays of Amylin (8–37) in RIN-m5F Cells in Absence or Presence of Aggregation Inhibitors

Rat insulinoma cells RIN-m5F were purchased from ATCC (American Type Culture Collection). The cytotoxic effect of human amylin (8–37) on RIN-m5F cells as well as the possible protective effect exerted by the amylin aggregation inhibitors analyzed *in vitro* were determined using the XTT reduction assay. Cells were cultured in 96-well plates at a density of 10,000 cells/well in fresh RPMI-1640 medium for 24 h. Amylin (8–37), previously aliquoted from 1,1,1,3,3,3-hexafluoro-2-propanol and lyophilized was reconstituted in 10 mM phosphate (pH 7.4), at a concentration of 500 µM. These fresh amylin solutions were then diluted to 75 µM in a culture medium in the presence of a 10 µM inhibitor (which provides 1% DMSO to the mixture) or in presence of just 1% DMSO as control (considered as 100% viability). After 48 h incubation, the culture medium was replaced with 100 µL of fresh RPMI-1640 medium, without phenol red, plus 50 µL of XTT reagent mixture following the manufacturer recommendations (Roche). Plates were then incubated at 37 °C for 4 h in a humidified atmosphere of 5% CO_2_ before measurement. Optical density was read at 450 nm with the reference filter set to 620 nm, using a spectrophotometric plate reader (Synergy, HT, Bio Tek, Winooski, VT, USA). Either triplicates or duplicates were used for each condition in seven independent experiments.

## 4. Conclusions

Using a combination of screening methods we have found that folic acid, quercetin, and benzbromarone inhibit amylin aggregation *in vitro*. Besides, quercetin partly protects cultured pancreatic insulinoma cells from the cytotoxic effect of amylin. A more detailed examination of a potential beneficial effect of quercetin in type II diabetes by reducing amylin aggregation *in vivo* and its concomitant oxidative damage in β cells may be timely.

## Figures and Tables

**Table 1:** Common names, systematic names, and chemical structures of three inhibitors of amylin (8–37) aggregation.

Aggregation Inhibitors	Structure
**Benzbromarone:** 3-(3,5-dibromo-4-hydroxybenzoyl)-2-ethylbenzofuran; (3,5-dibromo-4-hydroxyphenyl)(2-ethyl-1-benzofuran-3-yl)methanone	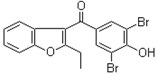
**Quercetin dihydrate:** 3,3′,4′,5,7-pentahydroxyflavone dihydrate; 2-(3,4-dihydroxyphenyl)-3,5,7-trihydroxy-4*H*-1-benzopyran-4-one dihydrate	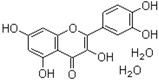
**Folic acid:** pteroyl-l-glutamic acid pteroyl-l-glutamate	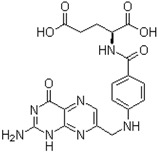

## Supplementary Materials

Supplementary materials can be found at http://www.mdpi.com/1422-0067/17/6/964/s1.
