# Supplementary material for: Benzbromarone, Quercetin, and Folic Acid Inhibit Amylin Aggregation
Source: Int J Mol Sci. 2016 Jun 18;17(6):964. doi: 10.3390/ijms17060964 (PMC4926496; doi:10.3390/ijms17060964)
Supplement: Supplementary file 1 [file ijms-17-00964-s001.pdf]

Supplementary Materials: Benzbromarone, Quercetin and Folic Acid Inhibit Amylin Aggregation

Laura C. López, Olga Varea, Susanna Navarro, José A. Carrodeguas, Natalia Sanchez de Groot, Salvador Ventura and Javier Sancho

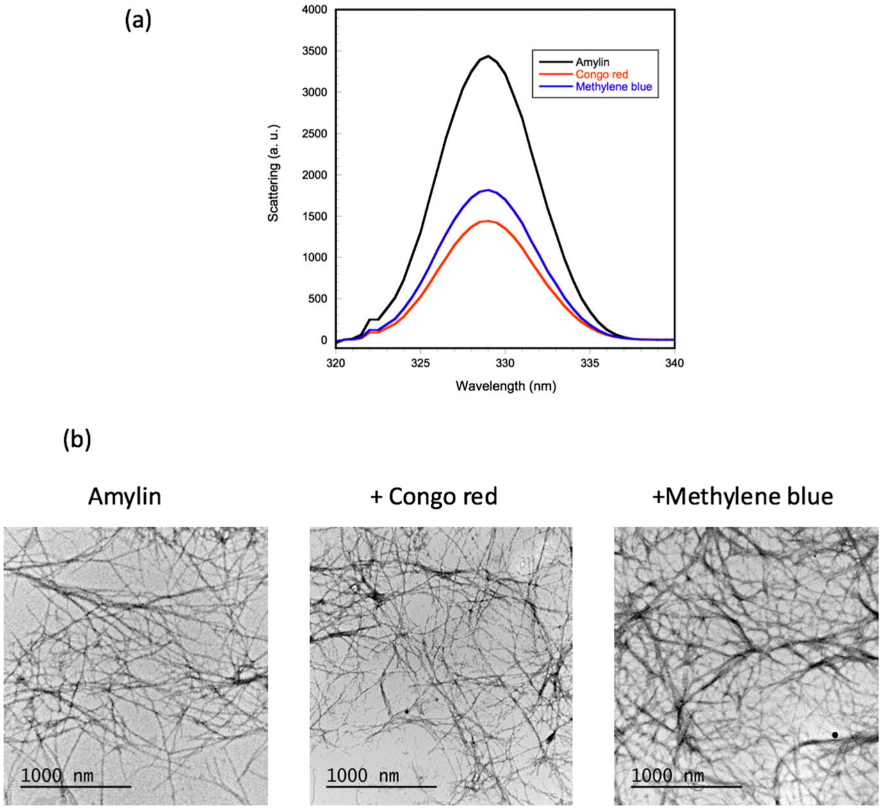

Figure S1. (**a**) Synchronous light scattering of 50 μM amylin (8–37) samples incubated alone or in the presence of Congo red and methylene blue; (**b**) Negatively stained transmission electron microscopy (TEM) photographs of the same solutions.

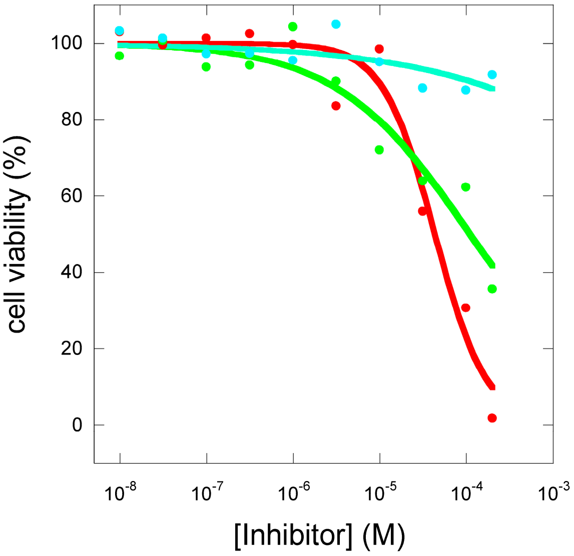

Figure S2. Cell viability of HeLa cells in the presence of different concentrations of inhibitory compounds. Red: benzbromarone; green: quercetin; blue: folic acid.
